# Inoculation and co-inoculation of two monosporic fungi onto surface-sterile blackberry fruits for quantification experiments

**DOI:** 10.1016/j.mex.2020.101092

**Published:** 2020-10-08

**Authors:** Irena Hilje-Rodríguez, Ramón Molina-Bravo

**Affiliations:** Escuela de Ciencias Agrarias, Universidad Nacional, Apartado Postal 86-3000, Heredia 40101, Costa Rica

**Keywords:** *Rubus adenotrichos*, Biological control, Phytopathology, Quantitative PCR

## Abstract

*Trichoderma* is used as a biocontrol agent against different plant pathogens in different crops. In Costa Rica, *Trichoderma* isolates from blackberry fruits (*Rubus adenotrichos* Schltdl.) have shown antagonism in laboratory and field trials against *Botrytis cinerea*. Quantifying fungal antagonistic activity directly on target organs or target tissues is of interest to estimate the performance of biocontrol agents. However, this is difficult due to the lack of visual manifestations of fungal structures. As part of a larger study to quantify antagonistic activity by quantitative PCR, we detail here a method to isolate and purify each fungus and then inoculate or co-inoculate them onto surface-sterilized blackberry fruits.

• A procedure to co-inoculate the surfaces of blackberry fruits with monosporic fungal suspensions for molecular analyses is described.

• The protocol described herein was implemented for subsequent qPCR analysis.

## Graphical abstract

**Figure:**
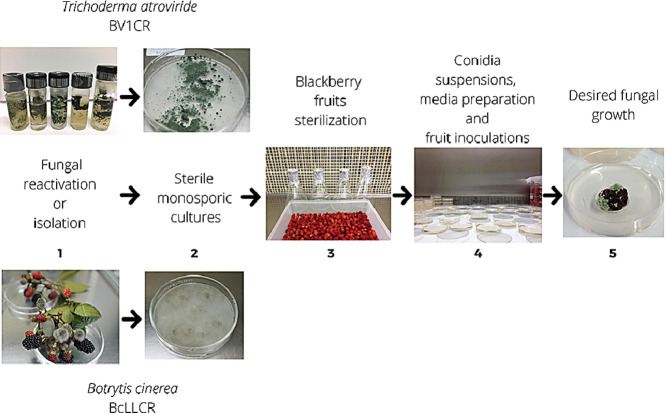

Specifications table

**Table:** 

Subject Area:	Agricultural and Biological Sciences
More specific subject area:	Biological control
Method name:	Co-inoculation of fungi onto surface-sterile blackberry fruits
Name and reference of original method:	N/A
Resource availability:	N/A

## *Method details

### Media preparation

For fungal cultures, potato dextrose agar (PDA) media (Oxoid Ltd., ThermoScientific™) was prepared by adding 39g of the selected media to 1L of ultrapure water. Complete medium dissolution was achieved by slowly mixing and heating the mixture. The prepared medium was sterilized by autoclaving at 121 °C for 20 min. Sterile PDA media was allowed to cool to around 50 °C and poured into sterile Petri dishes (200 × 15mm) (20 mL per plate) previously dripped with 25% lactic acid inside a laminar flow cabinet. Water agar (WA) (Oxoid Ltd., ThermoScientific™) media was prepared by adding 30g of agar to 1L of ultrapure water and autoclaved. For monosporic cultures and inoculation assays, prepared WA medium was poured into sterile Petri dishes (200 × 15mm and 60 × 15mm; 20 and 10 mL per plate respectively) previously dripped with 25% lactic acid. To immobilize berries, holes were punched in the center of the 60 × 15mm Petri dishes using a 10 mm width stainless steel cork borer.

### Fungal isolation and culture

*Botrytis cinerea* (BcLLCR) mycelia and conidia from infected blackberry fruits brought from the field [2] were isolated with a dissecting needle, placed over a microscope slide and observed under a light microscope for identification. Fungal tissues were cultured in Petri dishes with 20 mL PDA + 25% lactic acid. Plates were incubated at 25°C in the dark for at least 3 d. After that time, the isolated fungus was purified and recultured in fresh media by placing a portion of mycelium in sterile Petri dishes with PDA + 25% lactic acid. Plates were incubated at 25°C with an alternating photoperiod of 12 h until BcLLCR formed a lawn. Pure cultures of BcLLCR were later obtained by single spore isolation (monosporic cultures) using the methods described by Choi et al*.* [1]. Briefly, a conidia suspension (described below) of the cultured fungus was prepared and afterwards, was placed with a cotton swab on sterile Petri dishes with 20 mL WA. Plates were left overnight and spore germination was observed within 24 h. Germinating spores were individually selected with a dissecting needle and transferred onto sterile Petri dishes with PDA+ 25% lactic acid medium and grown at 25°C with a photoperiod of 12 h.

**Fig. 1:**
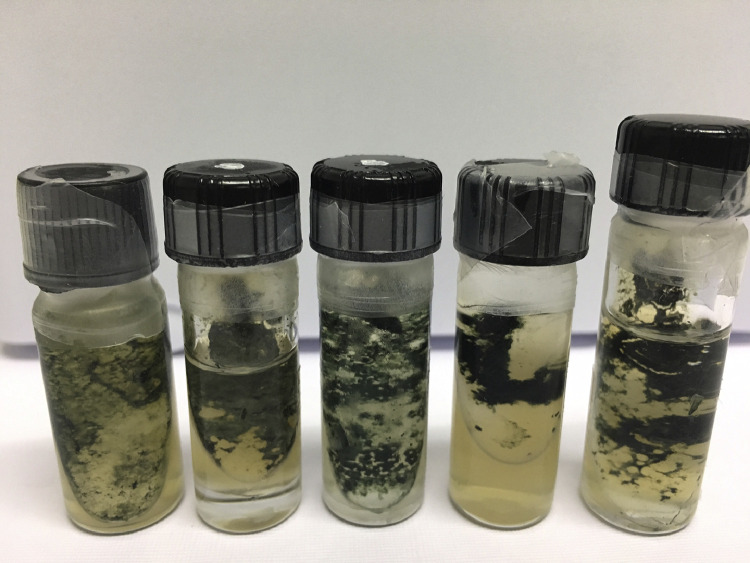
*Trichoderma atroviride* BV1CR used in the present study, preserved in vials with PDA + 25% lactic acid and sterilized mineral oil.

A *Trichoderma atroviride* isolate, BV1CR [2], was reactivated and cultured by following the methodology described above for *B. cinerea*. The fungal isolate was preserved at 4°C [3] in vials with PDA + 25% lactic acid and mineral oil (Fig. 1). For its reactivation, mineral oil was decanted and portions of fungal tissue were cultured in Petri dishes with 20 mL of PDA + 25% lactic acid. Pure cultures of BV1CR were obtained by single spore isolation (monosporic cultures) as described above for BcLLCR.

### Blackberry fruits sterilization

**Fig. 2:**
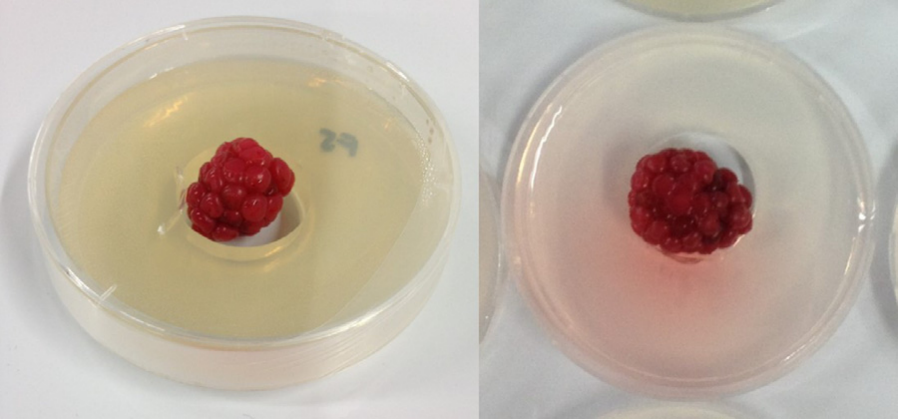
Healthy sterilized half-ripen blackberry fruits in Petri dishes (60 × 15mm) with 10 mL of water agar (WA). WA was punched with a 10 mm width stainless steel cork borer to immobilize berries.

Healthy half-ripen blackberries were wrapped and tied up in sterile gauze, placed in a sterile beaker and superficially disinfected by submersion in 75% ethanol for 30 s, 0.5% sodium hypochlorite (NaClO) for 1 min and rinsed three times in sterile distilled water for 1 min. Afterwards, fruits were placed on a sterile paper towel. Each sterilized fruit was placed in the center of a sterile Petri dish (60 × 15mm) containing bored WA media (Fig. 2).

### Conidia suspensions and dilution preparation

Conidia suspensions were prepared from monosporic cultures of each fungus (BcLLCR and BV1CR). To do so, conidia were collected with a sterile loop by scratching the plates’ media and placed inside a beaker with sterile distilled water. Beakers were shaken to separate spore aggregates. One drop of each conidia suspension was placed in an haemocytometer (Neubauer chamber) to estimate spore concentration. Once the number of spores was estimated, conidia suspensions were diluted and adjusted to 1 × 10^6^ conidia/mL with sterile distilled water.

### Fungal inoculations

**Fig. 3:**
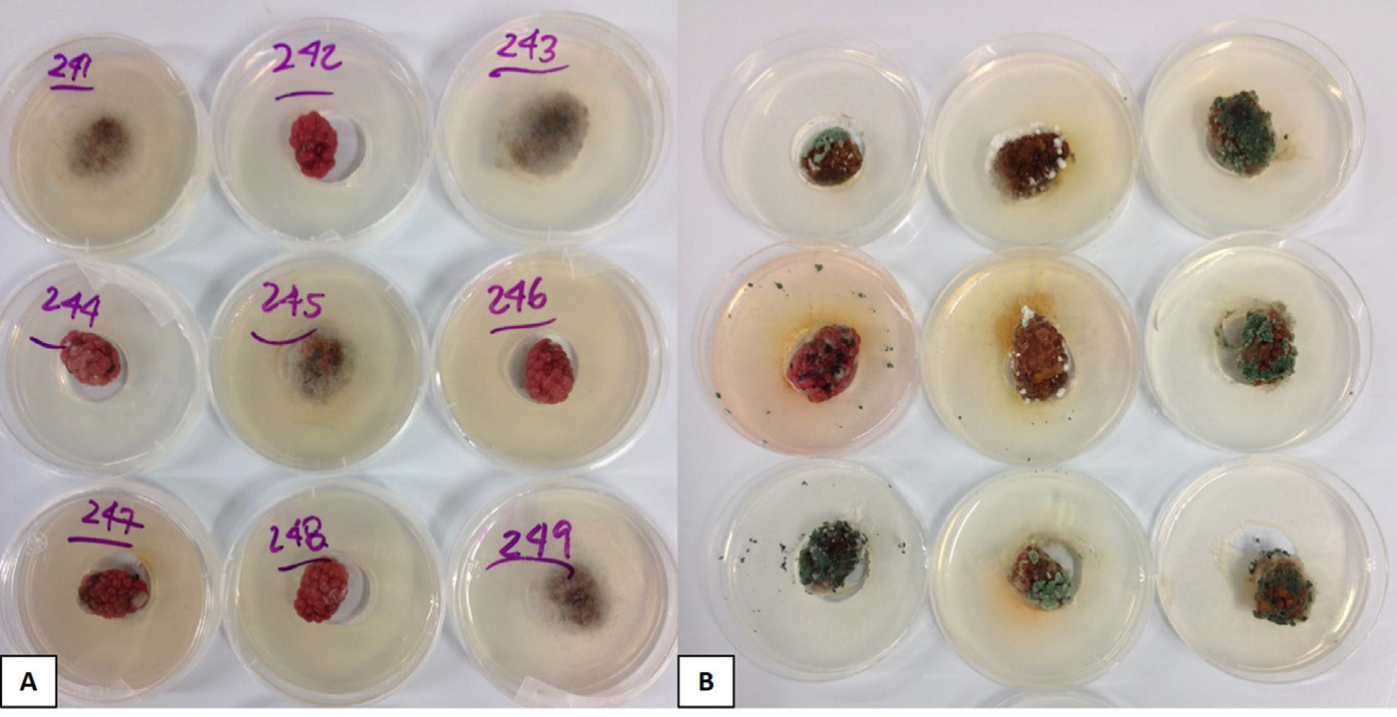
Inoculated blackberries with (A) ultrapure water (control treatment) and (B) *Botrytis cinerea* BcLLCR + *Trichoderma atroviride* BV1CR at day 10 after inoculation.

The sterilized blackberries were superficially inoculated with 20 µl of the conidial suspensions of BcLLCR and/or BV1CR at 1 × 10^6^ conidia/mL. As a control treatment, fruits were inoculated with ultrapure water. Twenty fruits were inoculated with each or a combination of fungal isolates (BcLLCR, BV1CR or BcLLCR + BV1CR) and incubated at 25°C with a photoperiod of 12h. Inoculated fruits were monitored for 10 d after inoculations (Fig. 3; [2]). Fruits showed the presence of other fungi growing from inside even in mock-water treatments, but to a lesser extent compared to inoculated and co-inoculated fungi (Fig. 3); approximately 50% less visual manifestations of fungal structures on berries and no manifestation on WA media. In inoculated and co-inoculated treatments, visual manifestations of non-inoculated fungi were rare and occurred to a lesser degree (Fig. 3B). This fungal growth did not interfere with qPCR analysis of BcLLCR and BV1CR. The interaction between these two fungi on blackberries was assessed by qPCR analysis using specific primer-probe sets that detected and quantified each organism and their growth separately in composite samples [2].

## Declaration of Competing Interest

The authors declare that they have no known competing financial interests or personal relationships that could have appeared to influence the work reported in this paper.

## References

[1] Choi Y.W., Hyde K.D., Ho W.H. (1999). Single spore isolation of fungi. Fungal Divers..

[2] Hilje-Rodríguez I., Albertazzi F.J., Rivera-Coto G., Molina-Bravo R. (2020). A multiplex qPCR TaqMan-assay to detect fungal antagonism between *Trichoderma atroviride* (Hypocreaceae) and *Botrytis cinerea* (Sclerotiniaceae) in blackberry fruits using a *de novo* tef1-α- and an IGS-sequence based probes. Biotechnol. Rep..

[3] Nakasone K.K., Peterson S.W., Jong S.C. (2004). Preservation and distribution of fungal cultures. Biodiversity of Fungi: Inventory and Monitoring Methods.

